# Applications of Support Vector Machine in Genomic Prediction in Pig and Maize Populations

**DOI:** 10.3389/fgene.2020.598318

**Published:** 2020-12-03

**Authors:** Wei Zhao, Xueshuang Lai, Dengying Liu, Zhenyang Zhang, Peipei Ma, Qishan Wang, Zhe Zhang, Yuchun Pan

**Affiliations:** ^1^Department of Animal Science, School of Agriculture and Biology, Shanghai Jiao Tong University, Shanghai, China; ^2^Department of Animal Science, College of Animal Science, Zhejiang University, Hangzhou, China

**Keywords:** genomic prediction, SVM, GBLUP, BayesR, molecular breeding

## Abstract

Genomic prediction (GP) has revolutionized animal and plant breeding. However, better statistical models that can improve the accuracy of GP are required. For this reason, in this study, we explored the genomic-based prediction performance of a popular machine learning method, the Support Vector Machine (SVM) model. We selected the most suitable kernel function and hyperparameters for the SVM model in eight published genomic data sets on pigs and maize. Next, we compared the SVM model with RBF and the linear kernel functions to the two most commonly used genome-enabled prediction models (GBLUP and BayesR) in terms of prediction accuracy, time, and the memory used. The results showed that the SVM model had the best prediction performance in two of the eight data sets, but in general, the predictions of both models were similar. In terms of time, the SVM model was better than BayesR but worse than GBLUP. In terms of memory, the SVM model was better than GBLUP and worse than BayesR in pig data but the same with BayesR in maize data. According to the results, SVM is a competitive method in animal and plant breeding, and there is no universal prediction model.

## Introduction

Breeding livestock and growing crops are the staples of agriculture. Since genomic prediction (GP) (Meuwissen et al., 2001) was proposed in 2001, it has significantly reduced the breeding time and costs involved with these aspects of agriculture (Resende et al., 2012). The rapid development of genotyping technologies has improved the availability of abundant single nucleotide polymorphisms (SNP), meaning GP is one of the most widely used methods in animal and plant breeding (Jiang, 2013; Jonas and Koning, 2015). GP has successfully improved rates of genetic gain (Bhat et al., 2016; Crossa et al., 2017).

Although GP has shown advantages in relation to various species such as dairy cattle (Schaeffer, 2006), pigs (Hickey et al., 2017), maize (Heffner et al., 2010; Albrecht et al., 2011), and the hybrid breeding of crops (Kadam et al., 2016), the accuracy of GP still needs to be improved. To predict breeding values more accurately, a variety of statistical genetics methods and prediction models have been developed (Yang et al., 2010; Bloom et al., 2013; Desta and Ortiz, 2014; Shigemizu et al., 2014). Most conventional models are linear as this approach is more efficient than non-linear models in terms of the non-additive genetic effect (González-Camacho et al., 2018) but some studies have shown that the non-linear model may perform better in some cases (Morota and Gianola, 2014).

The schema of predicting future breeding values based on information on training population falls into the scope of machine learning (ML). ML is the scientific study of algorithms and statistical models that computer systems use to learn from data (Samuel, 1959). ML has been used in many fields including personality recommendation systems, financial anti-fraud, speech recognition, natural language processing, machine translation, and image recognition, etc. (Makridakis et al., 2018).

The Support Vector Machine (SVM) is a well-known machine learning algorithm, which is a powerful method for classification and regression. Compared to the other ML methods, SVM is powerful at recognizing subtle patterns in complex data sets (Aruna and Rajagopalan, 2011). It uses multiple feature vectors to complete prediction by creating a decision boundary between two classes (Noble, 2006). SVM also has a strong and flexible ability to deal with all kinds of data due to various kernel functions. SVM is used to analyze a variety of complex biological data sets, including microarray expression profiles, DNA and protein sequences, protein-protein interaction networks, tandem mass spectra, etc. (Ahmadvand et al., 2017; Huang et al., 2017; Zhang et al., 2017).

Based on different kernel methods, SVM can also handle the non-linear relationship between phenotype and genome to some extent. Ornella et al. (2014) appraised six popular algorithms including SVM in wheat rust databases, and the authors recommend that the classification algorithms are competitive in plant breeding. Recently, González-Camacho et al. (2018) evaluated linear models, and several ML methods, such as random forest, SVM, and neural network in wheat rust data sets. They found that SVMs with linear kernels are superior in terms of GP (González-Camacho et al., 2018). Moreover, compared with SVM, neural network tuning is more complicated, time-consuming, and easy to overfit for data with more features. In the random forest algorithm, overfitting may occur when there is too much noise. These advantages mean SVM could be applied in animal and plant breeding more successfully.

Although SVM and other ML methods have been applied in many scientific and technological fields, it is still unclear whether these methods could outperform traditional statistical models in animal and plant breeding due to the fact that there is little empirical evidence on machine learning in this field. In most cases, conclusions were based on several or even single trait data, which has led to statistical significance and generalization of the results. Meanwhile, there were no benchmarks to compare the performances of ML methods with traditional methods (Makridakis et al., 2018). The performance of the different kernel functions implemented in SVM has rarely been compared in genomic prediction. In this paper, we compared SVM algorithms with two popular conventional GP models, GBLUP and BayesR, using different types of kernel functions. In addition to comparing the accuracy of the predictions, the actual application was also used as a standard. Therefore, the prediction performance of these three methods was compared in eight data sets on pigs and maize. In terms of time, memory and prediction accuracy were used as metrics.

## Materials and Methods

### Model Implementation

#### Genomic Best Linear Unbiased Predictor (GBLUP) Model

The GBLUP method was previously reported by Habier (Habier et al., 2007). It accounted for covariance between individuals using a genomic marker-based relationship matrix. The model is as follows:

(1) $$\begin{array}{c} \text{y}=\text{Xb}+\text{Zg}+\text{e} \end{array}$$

where ***y*** is a *n*×1 vector of response variable; ***X*** is a *n*×*p* design matrix relating the fixed effects to the response variable; ***b*** is a *p*×1 vector for the fixed effects. ***Z*** is a *n*×*q* design matrix for random effects; ***g*** is a *q*×1 vector of additive genetic effects for an individual, and ***e*** is a *n*×1 vector for the residual error. Furthermore, the random effects and the residual error are assumed to be normally distributed and mutually independent, i.e., $g∼N\,\left(0,G\,\sigma_{g}^{2}\right)$ and $e∼\text{N}\,\left(0,I\,\sigma_{e}^{2}\right)$, where $\sigma_{g}^{2}$ is additive genetic variance, $\sigma_{e}^{2}$ is residual variance. And ***G*** is the q×q genomic relationship matrix which can be calculated by the VanRaden method (VanRaden, 2008):

(2) $$\begin{array}{c} G=\frac{{\text{WW}}^{T}}{2\,\sum_{j=1}^{m}p_{j}\,\left(1-p_{j}\right)} \end{array}$$

Where each element of *W* is *W*_*ij*_ = *P*_*ij*_−2*p*_*j*_, *P*_*ij*_ is the SNP coded with 0, 1, 2 and *p*_*j*_ is the allele frequency at the *j*th marker.

#### BayesR Model

Compared with the GBLUP model that assumes all effects of markers drawn from the same normal distribution, BayesR assumes that the SNP effects are derived from a series of normal distributions, which are more in line with the actual situation. Some studies have proved that BayesR can get better results than GBLUP and other Bayes methods (Moser et al., 2015; Zeng and Zhou, 2017). The model is as follows:

(3) $$\begin{array}{c} \text{y}=\text{Xb}+\text{Z}\,\gamma +\text{e} \end{array}$$

Where ***y*** is a *n*×1 vector of response variable; ***X*** is a *n*×*p* design matrix relating the fixed effects to the response variable; ***b*** is a *p*×1 vector for the fixed effects. ***Z*** is a *n*×*m* design matrix allocating records to the marker effects; γ is a *m*×1 vector of SNP effects assumed SNP $\gamma_{i}∼N\,\left(0,\sigma_{i}^{2}\right)$, where the variance of the *i*th SNP effect had four possible values:

(4) $$\rho \,\left(\gamma |\pi ,\sigma_{\gamma }^{2}\right)=\pi_{1}\times N\,\left(0,0\times \sigma_{\gamma }^{2}\right)+\pi_{2}\times N\,\left(0,10^{-4}\times \sigma_{\gamma }^{2}\right)\begin{array}{c} +\pi_{3}\times N\,\left(0,10^{-3}\times \sigma_{\gamma }^{2}\right)+\pi_{4}\times N\,\left(0,10^{-2}\times \sigma_{\gamma }^{2}\right) \end{array}$$

Due to this equation, the model uses mixture distributions with SNP variances of 0, 0.0001, 0.001, and 0.01, so that the variance of the *i*th SNP has four possible values: $\sigma_{i\,1}^{2}=0$, $\sigma_{i\,2}^{2}=0.0001\times \sigma_{\gamma }^{2}$, $\sigma_{i\,3}^{2}=0.001\times \sigma_{\gamma }^{2}$, $\sigma_{i\,4}^{2}=0.01\times \sigma_{\gamma }^{2}$. The unknown parameters $\left(b,\pi ,\gamma ,\sigma_{\gamma }^{2},\sigma_{e}^{2}\right)$ are obtained through MCMC iterations.

#### Support Vector Machine

The Support Vector Machine, which was first proposed in the 1990s by Vapnik (Cortes and Vapnik, 1995), was used mostly in handling classification or regression problems. In this study, we used epsilon-support vector regression (Chang and Lin, 2011). To perform a non-linear regression, data were mapped into a higher dimensional space by kernel function (Hastie et al., 2009). Briefly, the model is:

(5) $$\begin{array}{cc} \text{y} & =\beta_{0}+{\text{f}}_{x}\,\left(\text{X}|\beta \right)+\text{e}\\ & =\beta_{0}+K\,\left(x,{\text{x}}^{T}\right)+\text{e} \end{array}$$

where **K**(*x*,*x*^*T*^) is an n×n kernel matrix, β is an n×1 vector (unknown). There are many different kernels, which are defined as Gaussian Kernel (Radial Basis Function, RBF):

(6) $$\begin{array}{c} {\text{K}}_{i\,j}\,\left(x_{i},x_{i}^{T}\right)=e\,x\,p\,\left[-\gamma \,\left(x_{i}-x_{j}\right)\,{\left(x_{i}-x_{j}\right)}^{T}\right] \end{array}$$

and Polynomial Kernel Function:

(7) $$\begin{array}{c} {\text{K}}_{i\,j}\,\left(x_{i},x_{i}^{T}\right)={\left(\gamma \,x_{i}\,x_{j}^{T}+r\right)}^{d} \end{array}$$

and Linear Kernel Function:

(8) $$\begin{array}{c} {\text{K}}_{i\,j}\,\left(x_{i},x_{i}^{T}\right)=x_{i}\,x_{j}^{T} \end{array}$$

and Sigmoid Kernel Function:

(9) $$\begin{array}{c} {\text{K}}_{i\,j}\,\left(x_{i},x_{i}^{T}\right)=t\,a\,n\,h\,\left(\gamma \,x_{i}\,x_{j}^{T}+r\right) \end{array}$$

In the process of solving SVM, eventually, it will be transformed into an optimization problem:

(10) $$\begin{array}{c} \min\frac{1}{2}\,||\omega ||+C\,\sum_{i=1}^{n}\varepsilon_{i} \end{array}$$

$$\begin{array}{ccc} \mathrm{subject}\,\mathrm{to} & & y_{i}\,\left(\omega^{T}\,x_{i}+b\right)\geq 1-\varepsilon_{i}\\ & & \varepsilon_{i}\geq 0,i=1,\ldots ,n \end{array}$$

Where ω is the hyperplane to be solved, ε_*i*_ is the regression loss for the ith sample point, and C is the penalty coefficient, which is the tolerance of the error. γ is a parameter of the RBF kernel function. The optimization of hyperparameters is a hard task to solve. We adopted a grid search which is one of the most frequently used methods for tuning hyperparameters, which can be found by trying all combinations and seeing which parameters work best.

### Genotypic and Phenotypic Data

In this study, three sets of data on maize, and five sets of data on pigs were used to evaluate the performances of different genomic prediction methods.

#### Pig Data Sets 1–5

One pig population used in this study from a pig farm of the Pig Improvement Company (PIC) (Cleveland et al., 2012). There are 3,534 samples genotyped by Illumina PorcineSNP60 chip and five traits. Phenotypes were corrected for fixed effects or were weighted progeny mean corrected phenotypes. The heritability (standard error) calculated by PBLUP for each trait was: T1 = 0.0773 (0.0272), T2 = 0.414 (0.0376), T3 = 0.3846 (0.0373), T4 = 0.3784 (0.0352), T5 = 0.445 (0.0358).

We discarded SNPs with more than 5% missing values, a minor allele frequency (MAF) < 0.01, or Hardy-Weinberg equilibrium (HWE) test *p* < 10^–6^. Because some individuals did not have all phenotypic data, the results in the number of individuals for each trait was different. For T1 (data set 1), T2 (data set 2), T3 (data set 3), T4 (data set 4), and T5 (data set 5), a total number of 45,025, 45,441, 44,190, 44,151, and 44,037 SNPs remained and were included in this study, respectively.

#### Maize Data Sets 6–8

One maize population investigated in this study is the NAM_US population. There are three flowering time traits in the NAM_US population, including days to anthesis (DTA, data set 6), days to silking (DTS, data set 7), and anthesis-silking interval (ASI, data set 8). All samples were planted under eight environments DTA, DTS, and ASI were measured and calculated as described by Buckler et al. (2009). Samples without phenotypic records, SNPs with MAF < 0.01, or SNPs with ambiguous position information were removed (Zhang et al., 2019). Finally, we obtained 4,328 samples with 564,692 markers.

### Method Implementation

The GBLUP method was performed by HIBLUP software^1^ in the R statistical software. BayesR (Moser et al., 2015) method was performed by BayesR software^2^ and SVM methods were fitted with the scikit-learn^3^ in python. These three models were selected and tested in both of the eight data sets, as described above.

It is important to select a suitable kernel function to construct an SVM prediction model with a favorable performance. The selection of the kernel function includes two parts: one is the choice of the kernel function type, and the other is the choice of the hyperparameters after the kernel function type has been determined. To select the SVM model that is most suitable for GS based on genomic information, we first tested the prediction performances of eight traits using four commonly used SVM models with different kernel functions in two populations. In this step, all SVM models used default parameters. Next in pig data sets, to get the bestγand C values in the SVM-RBF model, we first ran several SVM scenarios with different tuning parameters. Based on these runs, we implemented the grid search method with a full factorial design for the two parameters. For C we used 1–20 and for gamma we used 1×10^−1^, 1×10^−2^, 1×10^−3^, 1×10^−4^, 1×10^−5^, 1×10^−6^, 1×10^−7^, and 1×10^−8^. Therefore, 160 combinations were run for each pig data set. In maize data sets, to get the optimal C value in SVM-Linear, we selected from 1, 1×10^−1^, 1×10^−2^, 1×10^−3^, 1×10^−4^, 1×10^−5^, 1×10^−6^, 1×10^−7^, 1×10^−8^, and 1×10^−9^. The SVM-Linear model was constructed using LinearSVR function in scikit-learn which can greatly improve the operation speed and reduce memory consumption.

### Prediction Accuracy Evaluation

In this study, 10-fold cross validation was used to evaluate the prediction performances of each method. The original sample was randomly divided into 10 sub-samples and each sub-sample was used as the validation set and the other 9 sub-samples were used as the training set. The average of the 10 results was taken as the final predicted value. The prediction accuracy was measured with Pearson’s correlation coefficient between corrected phenotypes adjusted for all known non-genetic factors and predicted breeding values.

## Results

### Prediction Accuracies of SVM Models With Different Kernels

#### Pig Data Sets 1–5

**FIGURE 1:**
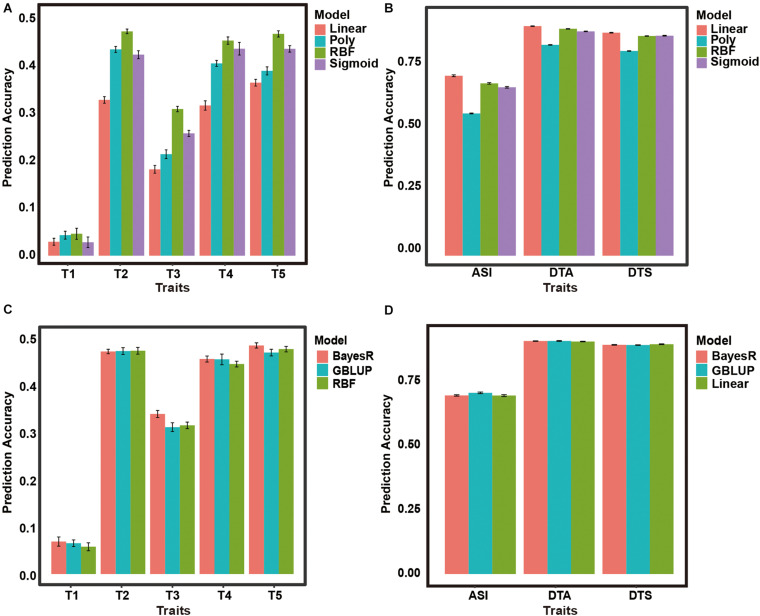
The prediction accuracies of GBLUP, BayesR, and SVM model in the pig and maize data sets. **(A)** Prediction accuracies of SVM models with different kernels in 5 pig data sets. **(B)** Prediction accuracies of SVM models with different kernels in 3 maize data sets. **(C)** Prediction accuracies of GBLUP, BayesR and SVM-RBF model in 5 pig data sets. **(D)** Prediction accuracies of GBLUP, BayesR and SVM-Linear model in 3 maize data sets.

Among the five traits, SVM-RBF had better performance. The accuracies of the SVM-sigmoid and SVM-poly models were similar. The SVM-linear model had the lowest accuracy among all the traits (Figure 1A). Therefore, in the five pig data sets, we choose the SVE-RBF model to further adjust the parameters for the next test.

#### Maize Data Sets 6–8

Among the three traits, the SVM-linear model had better performance, but the difference between the accuracy of the SVM-RBF model and the SVM-linear model was small. The SVM-Poly model had the lowest accuracy among all the traits (Figure 1B). It can be seen that for animal data and plant data, and even the different traits of the same species, different SVM models may have different performances.

### SVM Model Tuning

Based on the results above, we implemented the SVM-RBF model in the five pig data sets. We implemented the grid search method with a full factorial design with the two parameters. Of all the results, the highest prediction accuracy was obtained when C equals 2 and γequals 1×10^−5^ in T5. Therefore, we determined that the SVM-RBF model cooperated with this hyperparameter group to predict T5 traits. Using this method, we have separately selected the optimal parameter combinations for T1 – T4 traits, which are T1: *C=8* and γ = 1×10^−8^; T2: *C=2* and γ = 1×10^−6^; T3: *C=11* and γ = 1×10^−7^; T4: *C=14* and γ = 1×10^−6^.

Similarly, we implemented the SVM-Linear model in the three maize data sets. The optimal C value was selected from 10 candidate values. Of all the results, the highest prediction accuracy is obtained when C equals 1×10^−5^ among the three maize data sets.

### The Evaluation of SVM, GBLUP, and BayesR

Based on the parameter combinations described above, we implemented these three prediction models for the eight traits of pig data and maize data respectively. Meanwhile, the prediction accuracy, memory use, and issues such as whether it was time consuming were also evaluated and compared among the three models.

The prediction performance of the three models in the pig data sets across the three models generally showed similar performance (Figure 1C). The BayesR model had better performance in the three traits (T1, T3, T5) and the prediction accuracy ranged from 0.071 to 0.503. Pearson’s correlation coefficients of the GBLUP model ranged from 0.068 to 0.488 and GBLUP had the highest accuracy in T4. Under the SVM-RBF model, the prediction accuracy ranged from 0.060 to 0.495. Meanwhile, SVM-RBF had the highest accuracy in T2. However, the prediction accuracy of this model ranks second in three traits and the difference from the other models was small.

Similar to the pig data, there were nuances in performance among the three models. GBLUP has the highest accuracy in ASI, and the SVM-linear has the highest accuracy in DTS (Figure 1D). Meanwhile, the accuracy of these three models is almost the same in DTA.

In addition to prediction accuracy, we also compared the time and memory performance of the three models. For this study, we carried out all benchmarks on a single server equipped with 32 cores (Intel Xeon CPU E5-2620 v4 @ 2.10 GHz) and 64 GB memory, running only a single job at a time on the server. In terms of time consumption, GBLUP has an overwhelming advantage compared with the two data sets (Table 1). It only takes 1–2 min to complete a prediction calculation. The SVM-RBF model takes about 10 min to complete a prediction calculation for the pig datasets because the size is relatively small. Compared to the BayesR model, which takes 1.5 h, the SVM-RBF model still has a great advantage. Similarly, in the three maize datasets, SVM-Linear takes about 0.25 h to complete a prediction calculation, while the BayesR takes 16.8 h. In tuning progress, we used fivefold cross validation. In the five pig data sets, 160 combinations are equivalent to 800 predictions, but it can also be run with multithreading. When 10 threads are taken, it takes about 14 h. In terms of practical applications, once the tuning is completed, it cannot be carried out in the future. Adopting the same process for the three maize data sets, the tunning progress needs 50 predictions. When five threads are taken, it takes about 2.5 h. In conclusion, the three methods have a special advantage in different data sets and traits. In terms of time consumption, both SVM-RBF and SVM-Linear models have a great advantage over the BayesR model, but perform worse than GBLUP. In terms of memory, the SVM-RBF model was better than the GBLUP model but worse than BayesR. Both the SVM-Linear model and BayesR model had the same results, which are better than GBLUP. The results indicated that SVM is a competitive method in terms of genomic prediction.

**TABLE 1:** The performance of the three methods in terms of time and memory.

	PIC		NUM	
	Memory (GB)	Time *(h)*	Memory (GB)	Time (*h)*
GBLUP	5	0.01	33	0.03
SVM	4.2	0.16	12	0.25
BayesR	0.6	0.67	12	16.8

## Discussion

The objective of this study was to compare the classic machine learning model SVM with GBLUP and BayesR. In previous studies, most applications of SVM to animal and plant breeding focused on the evaluation of a certain kernel function in a certain trait in terms of prediction accuracy. For example, Ahmadi and Rodehutscord (2017) applied SVM with the RBF kernel function in predicting metabolizable energy in compound feeds for pigs. Montesinos-López et al. (2018) evaluated the performance of SVM-RBF in different traits of wheat data. It is important to correctly evaluate the performance of SVM models with different kernel functions using different animal and plant data sets and to compare them with conventional models. In this respect, we compared the performance of SVM with different kernel functions in eight different traits of pig and maize and evaluated different models in terms of prediction accuracy, the time it took to make the calculation, and memory use. Our results support the better application of the SVM method in animal and plant breeding through comprehensive comparison.

Four SVM models with different kernel functions were implemented for eight traits in pig and maize data sets. We found that SVM-RBF has higher prediction accuracy among four SVM models in pig data sets. The difference is that SVM-linear had higher prediction accuracy in maize data sets. Generally, the linear kernel function, with few parameters and a faster speed, is mainly used in linear separability. The RBF kernel function is mainly used in linear inseparability. It is worth noting that the accuracy of the linear kernel function is lower in the data on pigs, which was significantly different from that of the maize. The reason for this result is probably that the number of SNPs in the maize population is much larger than that in the pig population, which is more suited to linear kernel function fitting (Hsu et al., 2003). When the number of features is large, the linear kernel has an obvious speed advantage.

The main advantage of the Support Vector Machine models in evaluating breeding are: (1) that SVM models fit different functions and different types of data well, through different kernel functions; and (2), that SVM is more suitable for non-linear fitting. It can fit non-linear functions well by mapping data to high-dimensional space, whereas GBLUP is only suitable for a linear function. In genomic prediction, the SVM method is supposed to be better than any linear predictor if there are epistatic effects between markers (Morota and Gianola, 2014). However, there are some limitations for both SVM or other ML method modeling techniques. (1) Training an SVM model is more difficult because we need to select one suitable kernel function and test different combinations of hyperparameters corresponding to C and gamma, and the results are very dependent on these parameters (Cristianini and Shawe-Taylor, 2000). (2) Using the SVM method requires some programming experience and statistical knowledge, which may increase the threshold for using it. (3) The prediction accuracy of the SVM method is closely related to the combination of hyperparameters in different data, which makes the application of SVM in different data increase the time cost.

Based on the above results, SVM is a very competitive method in genomic prediction, which can bring alternative innovations for animal and plant breeding. It must be pointed out that SVM still has many limitations when it is applied in practice since it is a methodology and needs to be adjusted according to the actual situation. Researchers need to deeply understand the principles of SVM, spend time and experience encoding data, or optimizing the hyperparameters when applying it to a specific problem. These opportunities and challenges coexist, and SVM and other ML methods require further investigation in providing new pathways for the use and exploration of biological data.

## Conclusion

This study shows how the SVM model can be applied to genome prediction in animal and plant breeding. The results obtained by the SVM-RBF and SVM-linear model provide a computationally efficient approach with good prediction performance in GS. Our results show that RBF and linear kernel functions are suitable for phenotypic prediction based on genomic information. The SVM-RBF and SVM-linear model predictions produced very similar predictions to those of the GBLUP model and BayesR model, and, in some cases, outperformed the other two models. The disadvantage of SVM or both machine learning methods is that, to produce reasonable predictions they require a complex process of fine-tuning that is challenging since it is a scientific process that requires specialist knowledge along with qualitative reasoning and decision-making. In conclusion, the SVM method is a practical way of implementing genomic prediction.

## Data Availability Statement

Publicly available datasets were analyzed in this study. The datasets can be found at https://onlinelibrary.wiley.com/doi/full/10.1111/tpj.13174 (maize-NAM_US) and https://www.ncbi.nlm.nih.gov/pmc/articles/PMC3337471/bin/supp_2_4_429__index.html (pig).

## Author Contributions

YP and ZZ conceived the study. WZ, DL, and ZYZ wrote code, analyzed data, and drafted the manuscript. ZZ, PM, XL, and QW designed the research and revised the manuscript. All authors reviewed the manuscript for intellectual content and approved the final publication.

## Conflict of Interest

The authors declare that the research was conducted in the absence of any commercial or financial relationships that could be construed as a potential conflict of interest.

## Footnotes

1 https://hiblup.github.io/

2 http://cnsgenomics.com/software.html

3 https://scikit-learn.org/stable/index.html
